# Supplementary material for: pyAKI—An open source solution to automated acute kidney injury classification
Source: PLoS One. 2025 Jan 3;20(1):e0315325. doi: 10.1371/journal.pone.0315325 (PMC11698361; doi:10.1371/journal.pone.0315325)
Supplement: S1 File — The checklist that was used for the initiation of the participating physicians. (PDF) [file pone.0315325.s001.pdf]

pyAKI - Introduction and Initiation Meeting

Explanation of the Study

- [ ] Thorough explanation of the study concept, goals and experiment setup
    - [ ] Goal of the study: Implementing a tested and standardised toolbox for automated AKI stage classification according to KDIGO criteria.
    - [ ] Concept and Setup: Creating a gold standard validation data set to test on by letting pyAKI and junior physicianslabel the data and letting senior physicians validate both labels and resolve conflicts.

Reiteration on KDIGO Criteria

- [ ] Explain the "three way approach of KDIGO classification" -> Classification by urine output, serum creatinine elevation and presence of renal replacement therapy
    - [ ] explain the classification based on urine output:
        - [ ] a reduction in urine output below 0.5ml/kg/h for 6 to 12 hours is defined as AKI stage 1.
        - [ ] Reduction in urine output below 0.5ml/kg/h for more than 12 hours is defined as AKI stage 2.
        - [ ] Reduction of urine output below 0.3ml/kg/h for at least 24 hours or anuria for at least 12 hours is defined as AKI stage 3.
    - [ ] explain the classification based on serum creatinine:
        - [ ] absolute creatinine elevation:
            - [ ] Serum creatinine elevation of 0.3mg/dl against the baseline is defined as AKI stage 1.
            - [ ] Serum creatinine elevation over 4mg/dl is defined as AKI stage 3, irrespective of the baseline.
        - [ ] relative creatinine elevation:
            - [ ] An 1.5-1.9 fold increase relative to baseline is defined as AKI stage 1.
            - [ ] 2-2.9 fold increase is defined as AKI stage 2.
                - [ ] 3 fold increase is defined as AKI stage 3.
        - [ ] explain the baseline definition
            - [ ] currently, there is no clear definition of the baseline that should be used for serum creatinine determination
            - [ ] for this experiment, the lowest creatinine within the first seven days of available data is used as a baseline. In addition, the baseline always has to

lie before the current observation point. So future serum creatinine values cannot be used as a baseline, even when they lie within the seven day window. This of course only applies to the first seven days of evaluation, after that, the lowest value within the first seven days is used as baseline. (This represents the FIXED_MIN baseline creatinine method in pyAKI)

   - [ ] Final classification based on a maximum of the former classifications

Presentation of Example Data and Dataframe

- [ ] thorough presentation and explanation of the data on a sample data frame, including meaning of all columns.
   - [ ] Junior Physician: Dataframe containing only the raw data
   - [ ] Senior Physician: Dataframe containing the raw data and two deidentified columns with labels

Explanation on Workflow

Junior Physicians

- [ ] Label the data according to your best knowledge using the methods we just discussed here. Write your defined label into the appropriate column as a value between 0 and 3. At the end, assign a final AKI stage to the appropriate column.

Senior Physicians

- [ ] You see here the raw data with two columns of assigned labels of AKI stages. Validate each label based on your best knowledge and the definitions we just discussed and if the labels at an observation timepoint are not equal, resolve the conflict and assign a true label. Write a short comment who was right and what was the mistake in the wrong labelled column.

Room for Question Answering
