# Supplementary material for: pyAKI—An open source solution to automated acute kidney injury classification
Source: PLoS One. 2025 Jan 3;20(1):e0315325. doi: 10.1371/journal.pone.0315325 (PMC11698361; doi:10.1371/journal.pone.0315325)
Supplement: Attachment — Submitted filename: Reviewer Comments.docx [file pone.0315325.s002.docx]

**Reviewer Comments**:

Thank you for the opportunity to review this important and practical work. I commend the authors in aiming to address an overdue need to standardize AKI diagnosis across all the healthcare system.

**Abstract**:

(1) Authors state: “*a negative impact on workload and study quality*.” Workload for whom (clinicians, medical coders, patients)? Study quality-past and future universal AKI diagnostic criteria to be applied more accurately? Please edit for clarification
(2) Material and Methods, author’s state, “we **defined** a standardized data model.” Use of defined is misleading- I believe the intent is “we **constructed**” consider revision for clarity.
(3) Materials and Methods, authors state pyAKI’s performance was performed and later state internal validation as well but do not state here “how” validation was methodologically performance.
(4) Results, Accuracy of 1.0 was stated, what value or metric was used to state the performance result(s).
(5) Discussion, authors state, “it provides a standardized data model and a comprehensive solution for consistent diagnosis,” to be utilized where (ex: during hospitalization, outpatient and by whom (bedside clinicians in real-time, medical billing coders, researchers, etc.? Please add/edit for context/impact to field of science.

**Introduction**:

(1) “Acute kidney injury (AKI) is a common organ dysfunction in critically ill patients, affecting up to 50% of all intensive care unit (ICU) patients”1,2. “**Definitions for AKI changed over the years**”3,4. Incomplete sentence- please revise.
(2) “Recently, the definition of AKI has been made easier, by implementing the KDIGO-definition for AKI.” Please add context of what was made “easier” and continued challenges despite this to strengthen the ‘need’ for your proposed tool.
(3) “a pipeline implementation for cross-project application defining a common standard for AKI is lacking.” Please elaborate on the ‘negative research effects’ (i.e., consensus, accuracy, validity) this causes and “what” impact this has on data science and future tool development.
(4) “The entire software we developed is open source and available for download 1. The dataset generated for validation and the software implementation are licensed under an open-source license and publicly available 2.” References provided in manuscript do not correlate with statements proposed.

**Materials and Methods**:

(1) “Dialysis was also considered for classifying AKI. Any use of dialysis was classified as KDIGO stage 3. Definition of anuria varies in the literature.” Fragmented sentences, consider revising for clarity.
(2) “Users can decide for themselves whether an ideal body weight or adjusted body weight should be used.” Limitations of the Cockcroft-Gault equation and “which weight” should be used remains an area of controversy. The modified formula using adjusted body weight if a height is provided in the proposed tool is certainly beneficial to improve eGFR accuracy. However, please further describe how the eGFR accuracy may be influenced by this somewhere within the manuscript.
(3) Section 2.2, “As the input, the output also is formatted as a time series.” Please revise for clarification. Consider relocating to the first sentence of section 2.2 to state time series formatting for the input and output data values.
(4) “AKI stages are evaluated at each point in time, in the case of our validation experiment, meaning at each hour of the ICU stay.” Awkwardly stated, consider ‘In our validation experiment, AKI stages are assessed at each point in time, specifically at every hour during the ICU stay.’

2.3 **Software Development**:

(1) I cannot comment on this section as I am not qualified to evaluate.
    2.4 **Validation Data Set:**
(1) “14 patients that had an AKI and one patient not fulfilling KDIGO criteria to serve as control.” The extremely small sample size is a **major limitation** to serve as a control in the validation process. Understandably, this is due to the limitations of the MIMIC-IV dataset. However, I encourage the authors to consider additional mitigation strategies to improve the sample size to better support the validation accuracy. (Ex: synthetic knowledge synthesis strategies)

3.2 **Accuracy**:

(1) Please state what an ‘acceptable accuracy standard’ threshold is within the manuscript.
(2) “after comparing to the labels generated by the senior physicians is depicted in table 3.3”. I do not see a table labeled 3.3 in the manuscript (table 2?)

3.5 **MIMIC Comparison**:

(1) Table 3, for clarification, please depict “what the numeric values in the MIMIC and pyAKI columns are referring to.” (quantity of labels?)

**Discussion**:

(1) “PyAKI outperformed the accuracy of a group of physicians on the intern level.” Intern level has not been described; I’m assuming you are referring to junior physicians?
(2) “On one occasion, due to a transmission error, a patient was classified to not having AKI at all by the physician’s classification, when in fact, pyAKI and the experts classification agreed, that the patient had AKI. Such minor mistakes, especially due to wrong transmission of single-category AKI determination to the overall AKI stage are typical for human level performance on complex time series data and should be avoidable by using a software pipeline.” Is this necessary to state? Consider relocating to a ‘limitations’ section of the manuscript.
